# Biodegradation of pharmaceuticals in photobioreactors – a systematic literature review

**DOI:** 10.1080/21655979.2022.2036906

**Published:** 2022-02-08

**Authors:** Katarzyna Chojnacka, Dawid Skrzypczak, Grzegorz Izydorczyk, Katarzyna Mikula, Daniel Szopa, Konstantinos Moustakas, Anna Witek-Krowiak

**Affiliations:** a Department of Advanced Material Technologies, Faculty of Chemistry, Wrocław University of Science and Technology, Wrocław, Poland; b School of Chemical Engineering, National Technical University of Athens, Athens, Greece

**Keywords:** Algae, photodegradation, pharmaceuticals, micropollutants, wastewater treatment, biodegradation

## Abstract

This work is a systematic review that reports state-of-the-art in removal of pharmaceuticals from water and wastewater by photosynthetic organisms in photobioreactors. The PRISMA protocol-based review of the most recent literature data from the last 10 years (2011–2021) was reported. Articles were searched by the combination of the following keywords: photobioreactor, pharmaceuticals, drugs, hormones, antibiotics, biodegradation, removal, wastewater treatment. The review focuses on original research papers (not reviews), collected in 3 scientific databases: Scopus, Web of Knowledge, PubMed. The review considered the following factors: type of microorganisms, type of micropollutants removed, degradation efficiency and associated products, types of photosynthetic organisms and photobioreactor types. The conclusion from the systematic review is that the main factors that limit widespread pharmaceuticals removal in photobioreactors are high costs and the problem of low efficiency related with low concentrations of pharmaceuticals. The review indicated a need for further research in this area due to increasing amounts of metabolites in the food chain, such as p-aminophenol and estrone, which can cause harm to people and ichthyofauna. Pharmaceuticals removal can be improved by adapting the type of microorganism used to the type of contamination and implementing photoperiods, which increase the removal efficiency of e.g. sulfamethazine by up to 28%. In the future, it is necessary to search for new solutions in terms of the construction of photobioreactors, as well as for more effective species in terms of pharmaceuticals biodegradation that can survive the competition with other strains during water and wastewater treatment.

## GRAPHICAL ABSTRACT

**Figure:**
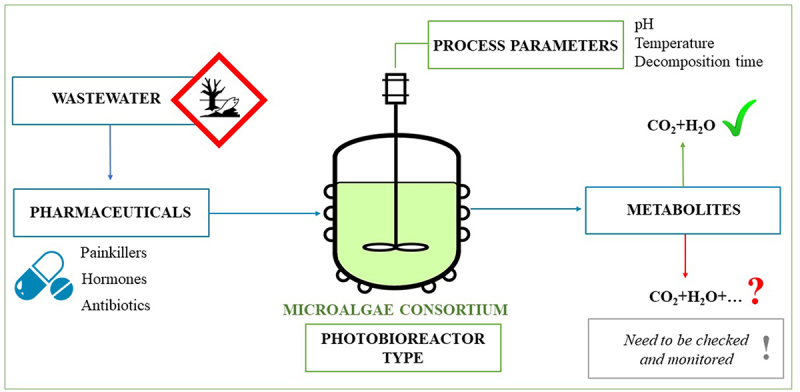

## 1. Introduction

Photosynthetic organisms have a unique ability to adapt to diversified environmental conditions and can biodegrade various organic compounds, including pharmaceuticals. Generally, these organisms are photoautotrophic, but they alter their metabolism under certain stress conditions (both abiotic and biotic) [1]. They have highly developed defense mechanisms and can transform metabolism from photoautotrophic to heterotrophic and mixotrophic pathways in response to environmental conditions, available light and organic substrates [2]. It is a unique characteristic of their metabolism that allows them to survive in environments strongly polluted also with xenobiotics, which is an expression of their adaptability [3].

Due to the progress in medicine, the amount of pharmaceuticals taken has risen exponentially. The most commonly used drugs are xenobiotic compounds, difficult in biodegradation, toxic with tendency of accumulation in the environment [4]. There are no standard water purification and wastewater treatment methodsfor these chemical compounds removal. Still, they have received increasing attention from authorities in recent years, prompting scientists to investigate new ways to effectively dispose of a wide range of pharmaceuticals [5]. Currently, there are many reviews on the effectiveness of removing these compounds from wastewater, which underline low efficiency, dependence on process conditions, a small range of applications, or a narrow spectrum of removed compounds 6. Only a few review articles have addressed the biodegradation of pharmaceuticals in photobioreactors in recent years. For this reason, it is essential to have a better understanding of biodegradation using microorganisms, closely related to pharmaceuticals disposal. An in-depth analysis of this topic will help toachieve the Global Sustainable Development Goals, particularly Clean Water and Sanitation (Goal 6) and Industry, Innovation and Infrastructure [Goal 9).

7,presented the review concerning the bioremediation of industrial and municipal wastewater by algae. The paper describes the removal of toxic metal ions and biogenic elements and focuses on microorganisms’ role in monitoring systems and their properties for deactivating substances harmful to the environment and humans. The work does not contain information on photobioreactors but only deals with biodegradation for removing mainly CO_2_ or inorganic compounds. This publication is devoted to biological wastewater treatment without explicitly mentioning products of the pharmaceutical origin or the active compounds of pharmaceuticals, which are the significant environmental hazard. The publication addresses microorganisms for the production of biofuels in wastewater treatment plants. It only explores the removal of the most frequently regulated chemical compounds. Our review does not cover those problems (7].

Enzymatic and microalgae-based methods to remove micropollutants from aquatic environments as a tertiary wastewater treatment technique were reviewed by 8. Recognition has been given to various contaminants: pesticides, personal care products and pharmaceuticals. The mechanisms of removing micropollutants from wastewater employing enzymes and a consortium of microalgae and bacteria were presented as degradation, immobilization, adsorption, bioaccumulation and co-metabolism. The review includes a list of methods and a description of process parameters. In this work, no spotlight was given to the construction of photobioreactors. Pharmaceuticals are presented as one example of micropollutants and microalgae as the exemplary class of organisms capable of biodegrading various micropollutants [8].

9,review the biodegradation of pharmaceuticals and personal care products through various microalgae strains. The authors highlight the role that microalgae play in the biodegradation of different classes of xenobiotics. Mechanisms were identified, while the scientists described the role of biosorption, bioaccumulation, biotransformation and biodegradation. However, photobioreactors and the aspects of their construction have not been presented for the biodegradation of micropollutants. The paper addresses the problem of the release of pharmaceuticals into the environment, resulting in antibiotic-resistant bacteria, which is a crucial element in the discussion of wastewater contamination with pharmaceuticals. Biodegradation has been described only in terms of the use of microalgae, without the participation of photolysis. This fact significantly distinguishes our review and shows the need to describe the topic of pharmaceuticals biodegradation in photobioreactors [9].

The review reports the design aspects of photobioreactors dedicated to microalgae cultivation with computational fluid dynamics in food production. This topic is not related to the scope of our review but contains helpful information on the design of a reactor working with a biological medium. The authors discussed modeling photobioreactors in open ponds and closed tanks, including heat and mass transfer, light transfer, growth kinetics, and hydrodynamics. They provide valuable knowledge on optimizing photobioreactors and analyses of the latest solutions in this technology, which is essential when starting research on this subject. The paper does not discuss photobioreactors in wastewater treatment, especially pharmaceuticals.

The review presented by 10,brings out the topic of photobioreactors for the energy industry, focusing on the biotechnological aspect of water purification. The authors determine the relationships between bioremediation and CO_2_ fixation in water treatment. The review presents biological treatment and the utilization of flue gas of thermal power plants, including photobioreactors, as possible solutions. The application of photobioreactors to remove pharmaceuticals was not mentioned because the focus was only on binding gases, pollutants formed in the thermal power plants without concentrating on biodegradation. Such information is not useful in determining the development paths for the biodegradation of pharmaceuticals in photobioreactors but only shows the possibilities of applying this type of solution in other industrial fields and describes the fundamentals of the design and basic principles of using such solutions on a larger scale [10].

11,presented various designs and applications of photobioreactors that use microalgae to remove pollutants from aquatic environments. Modeling of processes, technical problems in photobioreactor engineering, comparison of microalgae growth in various types of photobioreactors, comparison of the removal efficiency of multiple pollutants: nitrogen, phosphorus, BOD/COD, various organic contaminants, personal care products, and pharmaceuticals, was described. The review considers the impact of active compounds from pharmaceuticals on flora and fauna [11].

Only the last-mentioned review is closely related to the topic of the present work, which only focuses on the biodegradation of pharmaceuticals in photobioreactors by particular strains of microalgae. No papers devoted to this narrow subject have been found. There are no systematic reviews on the biodegradation of pharmaceuticals in photobioreactors. The last of the mentioned reports appeared in 2019. In the last 2 years, 21 original papers have been published with the keywords ‘*photobioreactors*’ and ‘*pharmaceuticals*’ (according to ISI Web of Science). This article reviews the latest papers on the biodegradation of pharmaceuticals in photobioreactors.

## 2. Materials and methods

The literature review was performed on 21/09/2021 based on 3 popular scientific databases: Scopus, Web of Knowledge, and PubMed. Using the algorithms offered by each database, a retrieval was performed for original research publications from the last 10 years (2011–2021) that included the words ‘photobioreactor’ and ‘biodegradation’ or ‘removal’ and ‘drug’ or ‘pharmaceuticals’ or ‘hormones’ or ‘antibiotics’ in the keywords, title or abstract. Applying the aforementioned algorithm in scientific databases: Scopus, Web of Knowledge and PubMed, 54, 76 and 5 articles were searched, respectively. Repetitive publications and papers in a language other than English, with no access to the full version, or incompatible topics were excluded. Twenty-one articles were used for the full analysis. The study was written following Preferred Reporting Items for Systematic Reviews and Meta-Analyses (PRISMA) statement guidelines for systematic reviews.

## 3. Results

**Figure 1.**
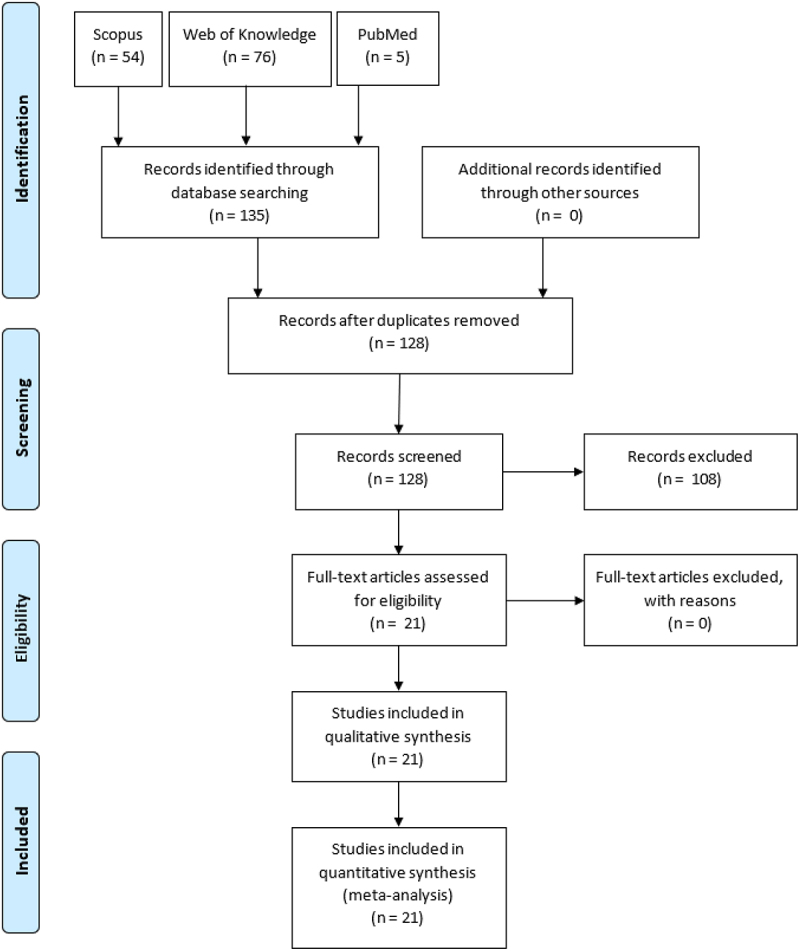
PRISMA flow diagram for pharmaceuticals biodegradation in photobioreactors.

The database searching results for the systematic review are shown in Figure 1. A total of 130 records were obtained for further consideration. In the first step, 2 repetitions were rejected. Subsequently, a more extensive screening was undertaken by checking the following parameters and rejecting publications in case of incompatibility: language (other than English), no access to the full version of the article, incompatible publication topic. Thus, 107 literature items were rejected, most of which were excluded due to incompatibility with the topic of this review. Twenty-one original research papers from the past 10 years with topics related to pharmaceuticals removal in photobioreactors using photosynthetic organisms were taken for complete review.

## 4. Discussion

### 4.1. Micropollutants

Pharmaceuticals have been of the primary concern by environmental organizations globally in recent years due to the growing consumption of pharmaceuticals, e.g., antibiotics, which from 2000 to 2015 amounted to about 21.1 to 34.8 trillion tons [12]. Currently, about 4,000 active compounds or their metabolites found in common pharmaceuticals have been identified 13, and no limits have been set for their discharge to the environment. The EU initiative is closest to regulating the presence of chemical compounds in water. The obligatory law does not include the active compounds of pharmaceuticals, they are included in the list for the observation. The EU is currently developing a single surveillance policy for these substances, but this requires more research in this area. In the case of pharmaceuticals, too little data on their toxic effects on the environment is available, with no comprehensive information on about 88% of drugs [5].

**Figure 2.**
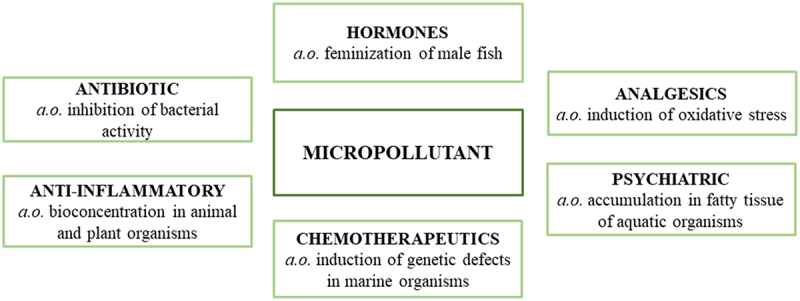
Types of micropollutants and their impact on the aquatic environment.

The most common pharmaceuticals discharged to aquatic environments are sulfonamides, tetracyclines, fluoroquinolones, macrolides, β-lactams, and aminoglycosides 6. These molecules have different effects on biota in the environment that can vary based on the category of a given pharmaceutical. The most frequently detected therapeutic groups are painkillers, hormones, antiparasitics, antibiotics [14], anti-carcinogenic [15], anti-diabetic, anti-convulsant, anti-fungal, antihistamine, and psychiatric drugs (Figure 2) [16]. These active substances may cause the following reactions to living organisms: organ damage, disturbances of breeding, hormone, growth, behavior as well as carcinogenicity, genotoxicity, mutagenicity. Those are just a couple of the numerous effects that can occur due to chronic exposure of living organisms to active substances of pharmaceuticals [4].

The concentration of pharmaceuticals in the output stream can reach values of mg/L, which shows the low efficiency of conventional treatment plants (T. ting Zhu et al., 2021), producing two types of pharmaceuticals: veterinary drugs and pharmaceuticals for humans. They affect many aspects of the ecosystem, including animal and fish farming, plant cultivation, and domestic animals used as organic fertilizers and applied directly on soil [4] (T. ting Zhu et al., 2021). These components penetrate through levels of soil into groundwater, surface water and thus are transferred to drinking water, plants, and animals. Finally, people intake metabolites of pharmaceuticals through the alimentary tract from those sources. Antibiotics are often thrown away due to the expiry of validity, for example. It is estimated that about 33% of the produced drugs are disposed of as waste. The annual consumption of pharmaceuticals depends on factors, such as seasons of the year, occurrences of allergy, flu, changes in temperature, or water level. Environmental pollution is also influenced by random events, such as downpours, floods, pandemics, migration, irrigation seasons related to the water management situation in a given year [5].

The high toxicity index of environmental contamination causes the formation of antibiotic-resistant bacteria. The spread of genes responsible for antibiotic resistance by microorganisms, plasmids, leads to modifications of bacteria (T. ting Zhu et al., 2021). According to recent studies, those microorganisms are present in input and output streams of sewage treatment plants. Pharmaceuticals are designed to inhibit specific molecules, i.e., have high chemical stability, slow degradation rate, and are used to elicit the desired response in humans and animals at low concentrations. Those properties are the main reason why pharmaceuticals are harmful to the environment [4].

According to 16, the amount of pharmaceuticals detected in surface waters varies depending on a given part of the world. More precisely, it depends on socio-economic development, which translates to the amount of antibiotics consumed. Research has shown the detection of 101 to 200 pharmaceuticals in highly developed countries like USA, Spain, UK, Germany [16].

The effect of antibiotics depends on many factors, such as toxicity, source of contamination, exposure period, degradation time, chemical stability, method of treatment in wastewater plants (type of process, which could have changed the chemical structure of a given compound and its decomposition to other more dangerous or more friendly/neutral metabolites [4, Zhu et al., 2021]. Highly devastating in aquatic environments are hormones. The studied effect concerns the feminization of male fish exposed to wastewater containing ethinylestradiol (EE2) [17]. The hormone is derived from contraceptives and is a synthetic estrogen. Even trace caoncentrations of this pharmaceutical cause feminization, thereby reducing the reproduction of the species [18]. Other adverse effects include long-term activity in the food web of water bodies or the deterioration of some populations. Painkillers and anti-inflammatories pose similar effects. The toxicity of anti-inflammatory drug residue (diclofenac) has been correlated with renal failure in animals [19].

Many countries have developed methods of controlling the amount of pharmaceuticals available for public use [16]. Still, no country has a comprehensive directive covering the entire life cycle of such products and their metabolites. Individual attempts to reduce their discharge include introducing a public taxation system to modernize and finance research solutions essential in reducing discharge of pharmaceuticals. The solution could be introducing lists containing information on the use of given pharmaceuticals in livestock breeding and for veterinary purposes or implementing bans on highly toxic drugs, e.g., in the EU, prohibiting growth hormones in the poultry industry [5].

Initiatives of different countries worldwide in counteracting the environmental threat from pharmaceuticals are set out in the United Nations 2030 Agenda and in the 2017 UN Assembly Ministerial Declaration, where activities related to testing resistance to antimicrobial drugs were agreed upon at the G-7/G-20 summits and by the World Health Organization. The general solution for the EU on this issue is Art. 8c of the Priority Substances Directive, which specifies that the future efforts should be comprehensive, i.e., covering not only the final stream from sewage treatment plants but also the modernization of production, the original composition of drugs, to create more environmentally friendly products and production methods [5].

Currently, there are no systematic methods of monitoring the concentration of pharmaceuticals in wastewater treatment plants; rather, screening controls in countries or plants specializing in, for example, in the production of these compounds [16]. The introduction of new monitoring methods is essential to define correctly the impact and relationship of pharmaceuticals with the environment. The application of combined monitoring is being considered. It consists of testing samples by liquid spectrophotometry with tandem mass spectrometry combined with microbial and plant growth inhibition tests. According to the publications, such a solution allows determining the concentration of active pharmaceutical compounds, their exact chemical structure, and chemical transformations [20]. It has been proven that some conventional methods used in wastewater treatment plants degrade these compounds into environmentally inert substances, converting them into another active form of pharmaceuticals (metabolites), which is harmful to the environment in the same or more significant way.

Table 1 presents a comparison of some of the commonly used wastewater treatment methods, with the specification of their application in the disposal of pharmaceuticals.

**Table 1.** Commonly used water treatment methods

Methods	Pharmaceutical Disposal Degree	Advantages	Disadvantages	Influencing factors	References
membrane bioreactor	Ibuprofen up to 99%, Carbamazepine up to 28%, Atenolol up to 96%	a small amount of sludge formed, possibility to modify, removal of high concentrations of pharmaceuticals, wide range of pharmaceuticals	membrane fouling, high cost, energy consumption	membrane type, physicochemical properties of micropollutants, operating condition,	[21]
conventional activated sludge	Ibuprofen up to 99%, Carbamazepine up to 25%, Atenolol up to 64%	cost, simple operation, simplicity in adjusting the parameters of the process	low efficiency in economic conditions, a small range of pharmaceuticals removed, requires a sludge recirculation system	hydrophobicity, properties of antibiotics, sludge properties, temperature, retention time,	[21]
sequencing batch reactor	Chiral pharmaceuticals (Alprenolol, Salbutamol, Norfluoxetine and so on), 48–63% on average	extensive modification possible, high loading rates, high tolerance to toxicity, simplicity in application and design	a low range of pharmaceuticals disposable	properties of antibiotics, HRT, SRT, sediment properties, temperature.	[22]
biological aerated filters system	Sulfamonomethoxine up to 99%, Sulfamethazine up to 23.7%, Amoxicillin up to 50.7%	low cost, energy consumption	susceptible to clogging, requires recirculation	HRT, pharmaceutical concentration	[23,24]
bioelectrochemical system	Ibuprofen up to 96.98%, Cefuroxime up to 100%	properties that stimulate the reaction, low cost, high yield, inhibit the production of toxic by-products, reduction of antibiotic resistance genes	high energy consumption,	electrochemical properties of pharmaceutical, electrodes, carbon source	[25,26]
constructed wetland	Carbamazepine up to 89.23–95.94%, Ibuprofen up to 89.50–94.73%, Sulfadiazine up to 67.20–93.68%	high efficiency, ecological, extensive range of removed compounds	possible use only at low concentrations of pharmaceuticals, depending on weather conditions	the water solubility of antibiotics, plant species, type of wetland, temperature, retention time,	[38]
biodegradation with UV irradiation	Clarithromycin up to 54–99%, Didofenac up to 40–99%, Lidocaine up to 94%	low cost, can be used as a pretreatment step, easy to apply	high cost, limited use only for removal photosensitive compounds, high energy consumption	UV dose, organic matter content, pharmaceutical chemical structure	[27]
biodegradation with ozone oxidation	Ibuprofen up to 95%, Metoprolol up to 60%, Sulfamethoxazole up to 95%	wide range of applications, there are no contraindications in the application	high cost, the difficulty of operations, process management	O_3_ concentration, pH,	[28]

In recent times, the impact of conventional wastewater treatment methods and their parameters on removing antibiotics, hormones, painkillers, bacteria, and genes that cause resistance to antibiotics has been investigated. The most commonly used processes are adsorption, membrane separation, coagulation, advanced oxidation, and biodegradation. In addition, the method adaptation and effectiveness are related to the chemical structure, hydrophobicity of pharmaceuticals, properties of, e.g., activated sludge, hydraulic time, oil retention, etc. [4, 26].

### 4.2. Mechanisms of pharmaceuticals biodegradation in photobioreactors and its efficiency

**Figure 3.**
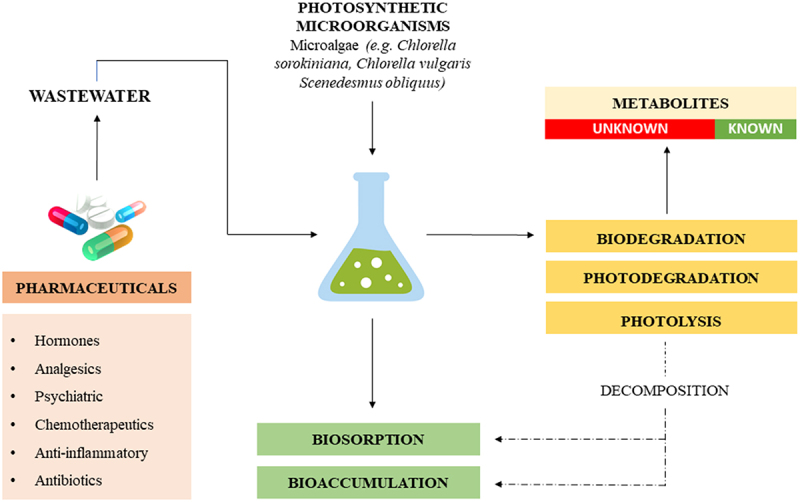
The concept of pharmaceuticals removal in photobioreactors.

The process of degrading pharmaceuticals in photobioreactors utilizes the potential of photosynthetic microorganisms. Through various mechanisms, drugs are biodegraded or bound by biomass (by biosorption or bioaccumulation), thus decreasing concentrations and removing micropollutants from wastewater (Figure 3). Photosynthesizing organisms can be used to degrade pharmaceuticals because, although photoautotrophic, they can switch the metabolism under specific stress conditions to heterotrophic or mixotrophic. This is a unique feature of their metabolism that allows surviving in environments heavily polluted with xenobiotics which is an expression of their adaptive abilities. The mechanisms described in the literature vary depending on the pharmaceutical substance being degraded and the microorganism. The results of the review are summarized in Table 2.

**Table 2.** Biodegradation efficiency of pharmaceuticals in a photobioreactor

Component	Initial concentration	Microorganism	Mechanism	Metabolites	Final removal (%)	Observations	References
17α-ethinylestradiol	1 mg/L	*Rhodopseudomonas palustris*	Biodegradation	estrogen	70%	Efficient biodegradation of ethinylestradiol in a hybrid photoassisted microbial fuel cell with simultaneous production of hydrogen as biofuel	[31]
Alfuzosin Atenolol Atracurium Bisoprolol Bupropion Citalopram Clarithromycine Metoprolol	-	Green algae *Dictyosphaerium*	Biodegradation	-	64,0 99,0 97,0 97,0 93,0 98,0 90,0 99,0	The analysis showed no relationship between pharmaceutical removal and light intensity reaching the water surface of the algal culture. Light intensity inside the culture had a significant effect on the reduction.	[42]
Amoxicillin	10–150 mg/L	*Chlorella* sp.	Mainly photodegradation by algae, also self-decomposition and adsorption	-	>99.4%	-	[32]
Carbamazepin Sulfamethoxazole Tramadol	0.2 − 1 mg/L	*Chaetoceros muelleri*	Biodegradation and bioaccumulation	-	64.8–70.2%	The concentration of pharmaceuticals and personal care products (PPCP) affects the microalgae *Chaetoceros muelleri* (above 40 mg/L causes a decrease in cell viability, chlorophyll and protein content)	[40]
Carbamazepine Ibuprofen Hydrochlorothiazide Gemfibrozil Bisphenol A	10 mg/L	Periphyton <94% *Phormidium*	Biodegradation	-	6,0–87,0	Higher hydraulic retention time increased the removal rate. Long exposure time negatively affected the efficiency of the reactor.	[44]
Carbamazepine Diclofenac Ibuprofen Lorazepam Oxazepam Diazepam	665.1 ng/L carbamazepine, 555.9 ng/L diclofenac, 101.4 ng/L ibuprofen, 0.38 ng/L diazepam	microalgae	Biodegradation, adsorption and photodegradation	-	11% carbamazepine, 52% diclofenac, 70% ibuprofen, 83% lorazepam, 71% oxazepam, 94% diazepam	Humic acids and the carbon exudates from the microalgae may increase the photodegradation of diazepam, transparency of the PBR tube material may limit light penetration and the photodegradation rate of photosensitive compounds	[41]
Ciprofloxacin	2 mg/L	Laboratory algal/bacterial culture	Photodegradation	-	84,0		[37]
Diclofenac	25 mg/L	*Chlorella sorokiniana, Chlorella vulgaris, Scenedesmus obliquus*	-	-	*S. obliquus* (99%), *C. vulgaris* (71%), *C. sorokiniana* (67%)	-	[29]
Diclofenac	25 mg/L	Microalgal *Chlorella sorokiniana, Chlorella vulgaris Scenedesmus obliquus,*	Biodegradation and adsorption	-	>79,0	The addition of diclofenac resulted in increased biomass growth due to the organic carbon source.	[48]
Diclofenac Ibuprofen Paracetamol Metoprolol Carbamazepine Trimethoprim	147 µg/L 317 µg/L 337 µg/L 181 µg/L 117 µg/L 202 µg/L	Microalgae *Chlorella sorokiniana*	Biodegradation and photolysis	-	60,0–100,0	Ibuprofen and diclofenac were removed photolytically, metoprolol and paracetamol by a combination of photolysis and biodegradation. No significant effect of contaminant concentration on biomass decline was observed.	[39]
Diclofenac Ibuprofen Carbamazepine	carbamazepine 510 ng/L diclofenac < 200 ng/L ibuprofen < 100 ng/L	Microalgae *Pediastrum* sp., *Chlorella* sp., *Scenedesmus* sp., cyanobacteria *Gloeothece* sp	Diclofenac, ibuprofen – photodegradation	-	Carbamazepine approx. 15 diclofenac 61 ibuprofen approx. 33	Reduction of biofouling and transparency of the photobioreactor polymer are important	[45]
Ketoprofen Naproxen Ibuprofen Acetaminophen Lorazepam Hydrochlorothiazide Ofloxacin Ciprofloxacin Diltiazem	472 ng/L 2945 ng/L 52091 ng/L 54294 ng/L 3696 ng/L 228 ng/L 65ng/L 2629 ng/L 1678 ng/L	Microalgal of lake water from Pantà de Can Borrell	Biodegradation and/or chemical transformation	-	36,2 10,2 98,5 99,2 57,2 44,3 67,7 47,6 77,3	The efficiency of the process is highly influenced by temperature and solar radiation. A high percentage of removal was obtained for anti-inflammatory drugs (98%).	[36]
Ketoprofen	2 mM	Microalgal *Chlorella* *Spirulina platensis* and bacterial consortium	Biodegradation	-	100,0	Chlorella microalgae had significantly higher resistance to ketoprofen concentration. They were found to biodegrade faster under dark conditions.	[47]
Ketoprofen, Paracetamol Aspirin	0,5 mM 0,5 mM 0,5 mM	Microalga *Chlorella* and bacterial consortium	Biodegradation	-	95,0	The best removal of analgesics was achieved with constant lighting.	[43]
Sulfadiazine Sulfamethazine Sulfamethoxazole	0,12 mg/L sulfadiazine, 0,046 mg/L sulfamethazine, 0,14 mg/L sulfamethoxazole	*Chlorella vulgaris*	Adsorption, bioaccumulation, and biodegradation	-	more than 30% for the algae batch culture in the flask; between 50–80% for continuous culture in BF-MPBR	Algal biofilms in the reactor reduce the frequency of microalgae collection	[35]
Sulfamethazine Sulfathiazole Sulfamethoxazole	-	Green microalgae *Botryococcus braunii*	Biodegradation	soluble microbial products and extracellular polymeric substances	38% Sulfamethazine, 53% Sulfathiazole and Sulfamethoxazole	*Botryococcus braunii* can biodegrade sulfonamides using Submerged Membrane Photobioreactors SMPBR with high efficiency	[34]
Sulfathiazole Trimethoprim Sulfamethoxazole	-	Microalgae	Biodegradation, adsorption and photodegradation	-	60–100%, with the exception of the antibiotics sulfamethoxazole (46%)	Reactor size, specific mixed cultures, temperatures and pH influence the productivity of PBR	[46]
Tetracycline antibiotics	0,25–30 mg/L	Mixed algae	-	-	-	Increased antibiotic concentration negatively affects algal biomass growth and photobioreactor performance. Eukaryotic algae were more sensitive to tetracycline antibiotics than cyanobacterial species.	[33]
β-estradiol	2 mg/l	Consortium (mainly *Chlorella* and *Pseudospongiococcum*)	Sorption, biodegradation, photodegradation and volatilization	estrone	55–100	Season of the year and daylight affect the efficiency	[30]

#### 4.2.1. Hormones

In the literature, 2 cases of biodegradation of hormones in photobioreactors have been reported. β-estradiol in the first phase is oxidized to estrone and furtherly fully mineralized to carbon dioxide and water. Other intermediate metabolites are not known. The first stage of metabolism was the fastest. In pilot studies, it was observed that the most significant degree of removal occurred during the first 13 h, particularly at night. Interestingly, estrone formation occurred under laboratory conditions in light and dark phases, indicating that this compound is not a photodegradation product. The pilot study proved that both the season and the degree of sunshine affect the biodegradation efficiency of estradiol, probably by changing the composition of the microbial consortium. The highest removal efficiency of estradiol was almost 94% under the most favorable weather conditions, while extreme process conditions (2°C temperature) allowed 50% degradation of the pollutant concentration [30]. To increase the biodegradation efficiency of 17α-ethinylestradiol, *Rhodopseudomonas palustris* was used in a hybrid microbial fuel cell with photoelectric support. After 16 days, 70% degradation of the resistant contraceptive component was observed. The experiments showed that adsorption and photodegradation are the dominating mechanism of ethinylestradiol removal. Only microbial degradation is a more likely pathway to reduce the concentration of the tested micropollutants [31].

#### 4.2.2. Antibiotics

The literature data in the analyzed range of years show 9 cases of biodegradation of antibiotics in single and in multicomponent systems (with different types of pharmaceuticals or other antibiotics). Amoxicillin degradation carried out with microalga *Chlorella* has very high efficiency, reaching more than 99% (in the range of initial concentrations corresponding to typical concentrations of this antibiotic in wastewater of 10–150 mg/L). Experiments were proposed to allow a detailed analysis of the removal mechanisms, which showed that biodegradation was the primary mechanism, especially during the first two hours of the process. Light-induced decomposition of the antibiotic and adsorption did not significantly affect the removal of amoxicillin (they were 3.8–26.4% and 1.8–12%, respectively). A mechanism for extracellular biodegradation by enzymes produced by the microalga and reactive oxygen species has been proposed [32]. Tetracycline was degraded using a consortium of green algae: *Chlorella* and *Pseudospongiococcum*. The study was conducted at a different concentration range of tetracycline 0.25 to 30 mg/L. It was observed that as the dose increased, the antibiotic showed a phytotoxicity effect affecting the inhibition of algae growth [33]. Sulfonamide antibiotics undergo degradation by the activity of microalgae. The literature describes the potential of *Chlorella vulgaris* and *Botryococcus braunii*. Studies have shown that the microalgae *Botryococcus braunii* can remove sulfonamides and release sulfur, nitrogen and phosphorus. The removal efficiencies of sulfamethazine and sulfathiazole were 38% and 53%, respectively [34]. After 12 days of an experiment for removal of sulfonamides from marine aquaculture wastewater using *Chlorella vulgaris*, decomposition of about 30% of sulfadiazine (SDZ), sulfamethazine (SMZ) and sulfamethoxazole (SMX) from wastewater were reported. These substances were mainly decomposed by microalgae (adsorption, bioaccumulation and biodegradation) supported by abiotic processes, such as hydrolysis and photolysis [35]. The biodegradation of ciprofloxacin has been reported twice in the literature. Hom-Diaz, with the team, presented the removal of ciprofloxacin from wastewater byusing a consortium based on microalgae and bacteria grown under laboratory conditions. It was found that photodegradation can provide a unique disposal of contaminants at lower concentrations and during daylight hours. At higher concentrations of the antibiotic at night, the removal mechanism of ciprofloxacin also includes a biosorption process on biomass. The metabolites that biodegradation products have not been investigated [36,37]. Furthermore, the biodegradation of clarithromycin using the green alga *Dictyosphaerium* has been described. The biodegradation efficiency was 90% [38].

#### 4.2.3. Psychiatric drugs

The most recent research articles reported biodegradation of psychotropic pharmaceuticals in 7 papers. Carbamazepine is most commonly degraded by biodegradation, supported by photodegradation, adsorption and bioaccumulation. These processes occur in the presence of plankton (*Chaetoceros muelleri*), periphyton, microalgae: *Pediastrum* sp., *Chlorella* sp., *Scenedesmus* sp., and the bacteria *Gloeothece* sp., *Proteobacteria, Bacilli* and *Fimbriiimonadia*. In only 2 cases, the removal efficiency exceeded 50% [39; 40]. In a study by 41, simultaneous degradation of diazepam, lorazepam and oxazepam was conducted. The high removal efficiency of diazepam (94%) may have been influenced by humic acids and carbonaceous secretions from microalgae, which enhance the photodegradation of this drug. The degradation product of diazepam and lorazepam is oxazepam, which is highly resistant to biodegradation (aerobic and anaerobic). Therefore, the mechanism responsible for concentration reduction was most likely adsorption onto microalgae biomass. Similar to lorazepam, except that photodegradation was an additional route of removal, which contributed to a yield of 83%. Using green algae, 93% of bupropion and 98% of citalopram were removed. Biodegradation was the dominant mechanism. The case also noted the effect of light intensity on biomass production and pharmaceutical removal rates [42].

#### 4.2.4. Analgesics, anti-inflammatories, chemotherapeutics and others

Other cases widely reported in the literature involve analgesics, anti-inflammatories, blood pressure and heart rate regulators, and inhibitors of prostatic hyperplasia. Decomposition of paracetamol leads to the formation of p-aminophenol, which shows more toxic effects than the decomposed substance. It is a monophenolic compound that can be easily biodegraded. Thus, paracetamol, which shows toxic properties through decomposition in photobioreactors, can pose an even greater threat to the environment. Using a consortium based on microalgae *Chlorella* and bacteria helps eliminate this problem. The use of the consortium and a continuous supply of air results in the complete degradation of both paracetamol and breakdown products. However, it should be noted that the hydraulic residence time must be extended to 4 days. Aspirin degrades to salicylic acid in the first step, while it completely degrades after 16 hours. The degradation was found to occur much faster under dark conditions [43]. The removal efficiency of hydrochlorothiazide, ibuprofen and gemfibrozil in periphyton photobioreactor showed a significant effect of the light cycle on the degradation of these compounds. Alternating light (12 h) and dark (12 h) cycles increased the removal efficiency of the tested Pharmaceuticals and Personal Care Products (PPCPs), despite a decrease in the amount of biomass produced with such photoperiod were observed. It is suspected that the growth of microorganisms with a higher capacity to biodegrade these compounds and a lower capacity to remove nutrients is promoted and associated with the presence of bacteria (presumably *Proteobacteria*) rather than algae [44]. Removal of ibuprofen and diclofenac was conducted in a hybrid system. Difficult-to-degrade compounds that do not undergo basic transformations (photo- and biodegradation) were removed in this system with low efficiency (ibuprofen – 33%). The high removal efficiency was achieved for diclofenac (over 60%), mainly due to photodegradation. In this case, adequate insolation is important, achieved in closed systems due to the good transparency of the photobioreactor material (polymers) and the reduction of biofouling [45]. Photobioreactors with periphyton do not show satisfactory removal efficiencies of ibuprofen (21%) and gemfibrozole (33%) due to the elevated pH of the environment. Above pH 8, the agents become fully deprotonated, which adversely affects sorption and its ability to pass through the microbial cell wall (cannot be metabolized) [44]. The presence of biocarbon can promote tramadol removal. The increased efficiency may be due to additional drug adsorption by biocarbon. Its presence may also promote biological degradation [40].

The removal of chemotherapeutics from wastewater has been described in two papers. Trimethoprim was removed using microalgae, yielding a 60–100% degradation. In both cases, biodegradation was supported by light scatter pattern [39,46]

### 4.3. Design strategies

#### 4.3.1. Photobioreactor types

The literature review has shown that different methods can solve the problem of pharmaceuticals decomposition in wastewater. However, studies conducted in recent years and focused on applying photobioreactors have proven the potential of different technological solutions. This diversity emerges mainly from the scale of the process (volume of the reactor), the substance to be decomposed, and the basic process parameters – pH, temperature or time of the decomposition. A summary of the most recent literature reports is summarized in Table 3.

**Table 3.** Types of photobioreactors for the removal of pharmaceuticals

Component	Scale	Photobioreactor type	Photobioreactor volume [dm^3^]	pH	T [°C]	Hydraulic retention time (HRT)	References
Tetracycline antibiotics	Laboratory	-	0.8	7.0	23	7 days	[33]
Carbamazepine Ibuprofen Hydrochlorothiazide Gemfibrozil Bisphenol A	Laboratory	Acrylic glass reactor	0.3	7.8	28	22 days	[44]
Carbamazepin Sulfamethoxazole Tramadol	Laboratory	Bubbling column	8	-	25	2.8 days	[40]
Diclofenac	Laboratory	Bubbling column	0.3	7.5	25	80 h	[48]
Diclofenac	Laboratory	Bubbling column	0.25	7.5	25	-	[29]
Ketoprofen	Laboratory	Flask	0.12	7.0	30	10 days	[47]
Diclofenac Ibuprofen Paracetamol Metoprolol Carbamazepine Trimethoprim	Laboratory	Flask	0.5	7.0	35,0	31 days	[40]
β-estradiol	Laboratory	Flask	0.25	-	23–27	-	[30]
Amoxicillin	Laboratory	Flask	0.25	-	25	-	[32]
Ketoprofen, Paracetamol Aspirin	Laboratory	Glass reactor	5	7.0	30	3–4 days	[43]
Ciprofloxacin	Laboratory	Glass reactor		7.0–10.0	20	3 days	[37]
Sulfadiazine Sulfamethazine Sulfamethoxazole	Laboratory	Microalgae biofilm membrane photobioreactor	1	-	26	1–2 days	[35]
β-estradiol	Pilot	Multitubular	1200	6.2–8.7	2.0–17.5	8–12 days	[30]
Antibiotics (9 types)	Pilot	Multitubular	1200	-	-	8–12 days	[49,50]
Ketoprofen Naproxen Ibuprofen Acetaminophen Lorazepam Hydrochlorothiazide Ofloxacin Ciprofloxacin Diltiazem	Pilot	Multitubular	1200	7.0–9.0	-	8 days 12 days	[36]
Alfuzosin Atenolol Atracurium Bisoprolol Bupropion Citalopram Clarithromycine Metoprolol	Full-scale	Open photobioreactor	650	8.3	10–32	7 days	[42]
Hydrochlorothiazide, ibuprofen, carbamazepine and gemfibrozil	Laboratory	Periphyton photobioreactor	30	7.8–8.9	28	2–4 days	[44]
17α-ethinylestradiol	Laboratory	Photobioreactor with hybrid photoassisted microbial fuel cell (h-PMFC)	0.50	-	-	16 days	[31]
Diclofenac, carbamazepine	Full-scale	Semi-closed (hybrid) tubular horizontal	8500	7.6–8.9	17.5	16 days	[45]
Sulfathiazole Trimethoprim Sulfamethoxazole	Pilot plant at demonstrative scale	Semi-closed horizontal tubular	3 ∙ 11,700	-	25	5 days	[46]
Carbamazepine Diclofenac Ibuprofen Lorazepam Oxazepam Diazepam	Full-scale	Semi-closed tubular horizontal	2 ∙ 11,700	8–10.5	24–25	5 days	[41]
Sulfamethazine Sulfathiazole Sulfamethoxazole	Laboratory	Submerged membrane photobioreactors	0.8	8.9	30	3–5 days	[34]

#### 4.3.2. Type of photobioreactor

The idea behind the construction of photobioreactors is to use the highest possible value of the ratio of the irradiated surface area to the reactor volume. Such construction ensures a shorter path of light penetration and, consequently, smaller differences in light intensities experienced by the cells of photosynthetic microorganisms during their growth. This translates into lower cell stress related to photoinhibition. It is then possible to obtain higher productivity of cell biomass growth and thus increase the rate and efficiency of photodegradation of pharmaceuticals. In some cases, photoinhibition and even light stress induction by high-frequency light flashes can initiate secondary metabolism in microalgae cells, which activates the mechanisms of photobiodegradation pharmaceuticals. Therefore, the design of the photobioreactor and the conditions for carrying out photobiodegradation should be individually selected for the strain to be cultivated and the compound to be removed.

Data obtained from the systematic literature review indicate that pharmaceuticals decomposition uses systems ranging in complexity from simple laboratory glassware to sophisticated membrane reactors. It is worth noting that the definition of the reactor type is often ambiguous and limited to the statement ‘glass reactor’ without specifying its shape.

Simple photobioreactors in the form of a flask or laboratory glassware not specified in shape were used in 7 publications. They were used for decomposition of analgesics [30,47], antibiotics, and hormones [30]. Thus, it was shown that there is no necessity to implement complex technological solutions. Nevertheless, a scale-up is required.

The bubbling column was used in 3 analyzed cases. The added value of such a solution is the gas stream introduced from the bottom of the reactor. This primarily ensures proper and uniform mixing, which is cost-effective than mechanical mixing. The dispersed gas provides favorable conditions for the growth of microorganisms and a homogenous distribution of nutrients to the cultivated microorganism and a homogeneous distribution of the distributed pharmaceutical in the volume of the reactor [48].

Three research publications describe the possibility of using a multi-tube photobioreactor, allowing all types of pharmaceutical degradation. The wastewater treatment time to remove micropollutants in such a technological solution is from 8 to 12 days [30]. A closed multi-tube photobioreactor with microalgae was used to remove pharmaceuticals from municipal wastewater [40]. The reactor was made of polypropylene and polyethylene. Distribution chambers were placed at the ends of the tubes to distribute the culture between the tubes evenly. Additionally, a paddlewheel made of polypropylene was placed in one of the distribution chambers to provide biomass aeration. This solution allows the biodegradation of large molecular pharmaceuticals to be studied simultaneously, as demonstrated for 9 antibiotics [49]. Multi-tube systems are increasingly appreciated as large-scale solutions because, due to their design, they provide a large volume with equal conditions for microbial growth.

Biodegradation of pharmaceuticals using semi-closed tubular horizontal photobioreactors has been described 3 times in the literature. These are open and closed systems consisting of open polypropylene tanks connected by tubes, also equipped with water level regulation, ensuring the gravitational flow of liquid between tanks [46]. The described method of conducting the process allows for the degradation of multiple pharmaceuticals simultaneously [45].

The potential of membrane photobioreactors has been demonstrated in 3 publications. Membrane photobioreactors are effective devices for the simultaneous separation of microorganisms on membranes. In 32,experiments, an ultrafiltration membrane with a pore size of 0.1 µm was applied with a low transmembrane pressure of less than 2 kPa and a permeate flux of about 3 LMH [32]. The mentioned type of reactor was also implemented in the degradation of sulfonamides from livestock wastewater. The glass bottle reactor used an immersion capillary membrane with a pore size of 0.5 µm [34]. A more advanced type is a biofilm membrane photobioreactor. This arrangement allows for continuous flow and the ability to separate algae by filtration of the membrane module. In conventional batch cultures, the separation of suspended algal cells arises and the methods used (membrane separation or flocculation) are time-consuming and expensive [35]. Biofilms reduce the frequency of algae collection, which significantly reduces process costs [1].

A hybrid microbial photoassisted fuel cell was implemented to remove ethinylestradiol, a component of oral contraception. Duran bottles with a working volume of 400 mL were used for laboratory testing. The bottom and top of the PBR consists of an electrochemical cell and membrane electrode assembly. This has the advantage of efficient ethinylestradiol removal and biofuel production in the form of hydrogen from the metabolic processes of aromatic compounds present in the wastewater [31].

Some cases also describe the possibility of application of reactors with periphyton [44] or open reactors [42]. The construction of such solutions requires the use of materials that allow light to reach from above and from the side and bottom of the reactor. This ensures a more intensive growth of microorganisms and, therefore, a higher efficiency of biodegradation of pharmaceuticals.

#### 4.3.3. Process scale

The literature review revealed that as many as 16 studies were conducted at the laboratory scale (0.3–30 L). The remaining 7 involved biodegradation of pharmaceuticals at pilot (1200–35,100 L) or full scale (650–23,400 L), but it can be noted that there is no unification in the size of these scales. The dominance of laboratory scale shows that the biodegradation of pharmaceuticals is relatively new, which needs to be continued and expanded in increased scale.

#### 4.3.4. Other process parameters

Photobiodegradation of pharmaceuticals, based on the literature, is usually carried out at a pH close to neutral or alkaline, in the range of 6.2–10.0 [30,37]. The pH value combined with temperature, which on average oscillates in the range of room temperature (20–25°C), allows for proper growth of microorganisms and decomposition of micropollutants, which mostly decompose at alkaline pH. The literature review has also shown the possibility of conducting the process at reduced or elevated temperatures. An increase in temperature significantly affects the efficiency of microbial biomass growth [34].

The cultivation of microorganisms in photobioreactors is usually carried out with a photoperiod of 12:12 h (day:night), with greater biomass growth being achieved with higher light intensity as long as it does not cause phytotoxicity [48]. Variations in the diurnal rhythm of solar radiation affect the performance as no photosynthetic activity and low pH values were observed at night [37]. This was confirmed in 34, where the applied photoperiod system was more effective, with yields 13 to 28% higher for sulfamethazine than continuous irradiation [34]. Research also pays attention to the insolation of photobioreactors, especially in full-scale biodegradation cases. A strong correlation between algal biomass and insolation has been observed. 45,conducted their research in the late winter and early spring, achieving higher yields in the spring, which was associated with an increase in day length (as well as temperature) [45].

Hydraulic retention time (HRT) was selected individually by the authors of various studies to the applied methodology: the decomposed pharmaceutical, the applied reactor and the process parameters: pH, temperature and photoperiod. The shortest biodegradation time was described in the study on Amoxicillin degradation and amounted to 12 h [32], the longest one amounting to 22 days in the case of Carbamazepine and Ibuprofen, among others [44].

Analysis of literature data showed that other substances could assist the biodegradation process of pharmaceuticals. 40,showed that the presence of biocarbon increases the removal efficiency of micropollutants [40]. 44,demonstrated that water from a eutrophic lake could be used as inoculum for periphyton culture [44]. In the work of 42, it was proved that during the biodegradation of pharmaceuticals in a bioreactor, the exhaust gas treatment process could be carried out simultaneously [43].

#### 4.3.5. Technological barriers – critical discussion

**Figure 4.**
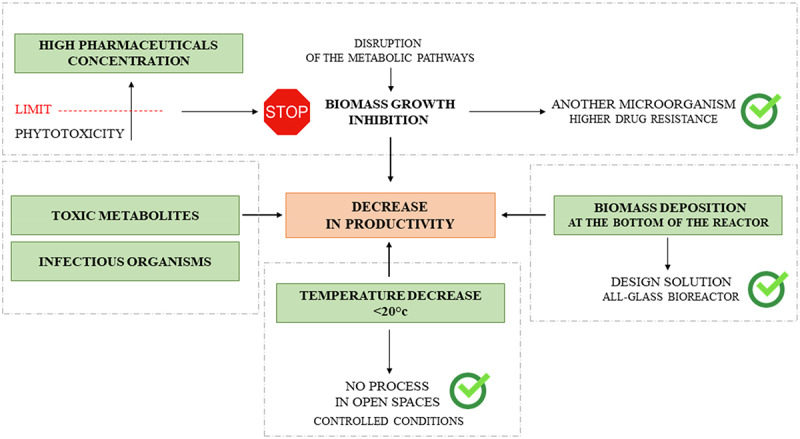
Technological barriers associated with different process parameters of pharmaceuticals degradation in photobioreactors.

A literature analysis of the biodegradation of pharmaceuticals in photobioreactors has highlighted several technological barriers associated with different process parameters (Figure 4). Their presence directly leads to a decrease in the efficiency of micropollutants degradation. This means that the issue of biodegradation requires further study and extension.

The most serious problem seems to be too high a concentration of pharmaceuticals. This leads to inhibition of biomass growth and, therefore, the efficiency of pharmaceuticals decomposition. This is associated with phytotoxicity effects due to disruption of microorganisms’ metabolic pathways, which has been demonstrated in several studies [33,40]. The problem can be solved by introducing another microorganism into the bioreactor that will show drug resistance, growth at a high rate and degrade pharmaceuticals with high efficiency.

Metabolites formed during biodegradation can also be toxic. Decomposition of paracetamol, resulting in the production of p-aminophenol shows more toxic effects than the decomposed substance [43]. On the other hand, biodegradation of β-estradiol leads to estrone, which is also a hormone. Its accumulation in the food chain can result in human metabolic disorders [30]. However, it is noteworthy that only a small part of the publication shows which metabolites are formed by biodegradation. This is a critical parameter that determines the effectiveness of biodegradation.

The decomposition of micropollutants is also limited by the process conditions – temperature and insolation/light. It has been shown that the efficiency of the process is higher in spring than in winter, which is related to temperature and day length [30]. It should be noted that the literature describes the issue of photoperiod multi-directionally. In his study, Ismail and his team presented two extremes: constant light is best, but on the other hand, processes happen faster under dark conditions [43,47]. On the other hand, 44,demonstrated that cycle variability is best. The variability of the sites is due to the use of different microorganisms in the study. Therefore further studies are required regarding the behavior of the microorganism and the parameters limiting its growth [44].

Insolation is also a key parameter in periphyton photobioreactors, where the biomass of microorganisms is deposited on the bottom [44], and open bioreactors, where microorganisms can form thick, dense layers on the upper surface [42]. Design solutions solve these problems. In the first case, the possibility of building an all-glass bioreactor is used, preferably placed on an elevation in the form of a frame, which will provide access to light from all sides. The second case requires mechanical mixing or a relatively strong barbotage, ensuring the circulation of biomass between zones with and without access to light.

There are several additional barriers to implementing the discussed technology on an industrial scale. It is worth mentioning the temperature requirements of algae culture, preferably above 20°C, significantly limiting the application in the winter season in an open area. Another critical parameter is the processing time, which ranges from a few days to tens of days, which is problematic when considering the amount of wastewater generated daily requiring treatment. Microorganisms typically work in consortia that collaborate in biological equilibrium. Sometimes this equilibrium can be disturbed by infectious organisms, which can cause adverse effects such as biomass degradation.

## 5. Future perspectives

Available knowledge and technological solutions show that pharmaceuticals decomposition in bioreactors based on different microorganisms can have high efficiency. Many issues need to be clarified. Perhaps not technical, but a serious barrier also arises from the lack of knowledge of the mechanism of degradation of pharmaceuticals by a given microorganism. Literature data indicate the possibility of co-occurrence of different pathways of micropollutant photobiodegradation: biodegradation, photolysis, photodegradation, biosorption, bioaccumulation or adsorption. The knowledge of the relation: pharmaceutical – microorganism – mechanism would allow modeling the process in detail to maximize its efficiency, in the shortest possible time and the simplest possible technological and structural solution.

Process modeling should also include in its scope the evaluation of metabolites of pharmaceutical biodegradation and their toxicity. The scarcity of information on this topic points out the way forward, as existing reports indicate the potential for forming other hazardous compounds, the concentrations of which should be monitored and effectively reduced. Micropollutants, although present in sewage in trace amounts, can impair the reproduction of aquatic organisms (especially hormones), inhibit their growth (especially antibiotics), and accumulate in the food chain (biomagnification) and eventually be taken up by humans, which in the case of low but permanent exposure (chronic exposure) can lead to hormonal and metabolic disorders or even drug resistance.

Pharmaceutical biodegradation in photobioreactors is relatively a new topic. The holistic approach taking into account all parameters and broadening the group of investigated drugs would allow developing an effective strategy to eliminate micropollutants from the environment.

## 6. Conclusions

The topic discussed in the review can contribute to the achievement of Global Sustainable Development Goals, including Clean Water and Sanitation (micropollutants removal) and Industry, Innovation and Infrastructure (modern design solutions). The review presented various technological methods for the removal of pharmaceuticals using a range of microorganisms and identifying the critical parameters of their removal processes. Technological barriers and shortcomings were also highlighted: the lack of validated metabolite analytical methods, the influence of sunlight/exposure on the growth of microorganisms, the need to adapt the type of reactor to the microorganism used, the mechanism of pharmaceuticals degradation and toxicity of the metabolites. The issue of drug resistance and pharmaceuticals activity on the environment requires the increased use of microorganisms to remove pharmaceuticals from wastewater and surface waters. This could be a way to eliminate these pollutants from the food chain and protect people and ichthyofauna.

Micropollutants (pharmaceuticals) occur in low concentrations in the environment and this poses problem with routine analysis of these compounds and their metabolites. Despite their low concentration, they exhibit a negative effect on organisms, which indicates the need to expand research in their removal.
